# Impact of different ground-based microgravity models on human sensorimotor system

**DOI:** 10.3389/fphys.2023.1085545

**Published:** 2023-02-15

**Authors:** Alina Saveko, Maria Bekreneva, Ivan Ponomarev, Inna Zelenskaya, Alexandra Riabova, Tatiana Shigueva, Vladimir Kitov, Nelly Abu Sheli, Inna Nosikova, Ilya Rukavishnikov, Dimitry Sayenko, Elena Tomilovskaya

**Affiliations:** Russian Federation State Scientific Center—Institute of Biomedical Problems of the Russian Academy of Sciences, Moscow, Russia

**Keywords:** groundbased observations, dry immersion (DI), bed rest, parabolic flight, space flight, immobilization, anti-orthostatic hypokinesia, suspension

## Abstract

This review includes current and updated information about various ground-based microgravity models and their impact on the human sensorimotor system. All known models of microgravity are imperfect in a simulation of the physiological effects of microgravity but have their advantages and disadvantages. This review points out that understanding the role of gravity in motion control requires consideration of data from different environments and in various contexts. The compiled information can be helpful to researchers to effectively plan experiments using ground-based models of the effects of space flight, depending on the problem posed.

## 1 Introduction

Gravity is an important factor that shaped life on Earth. Space flights (SFs) made it possible to study the reaction of living systems to changes in gravity. Despite this, even after 60 years of human space exploration, many physiological mechanisms of adaptation to microgravity remain unknown. Few opportunities for SFs, high cost, and long intervals between launches complicate space experiments with human participation. Ground-based models of key SF factors make it possible to design, replicate, or confirm studies under controlled conditions to draw informed conclusions about the effect of microgravity on the human body (Reynolds and Shelhamer, 2020). Also, ground-based models are necessary to predict health risks and to develop and select means to prevent the adverse effects of microgravity.

The sensorimotor system includes all afferent, efferent, and central components of integration and processing. Although these processes may impact cognition and emotion contribute, our work focus on the motor control system.

Among the factors that can cause the negative impact of SF on the human sensorimotor system, changes in the activity of the sensory systems occupy an important place. In weightlessness, the activity of afferent signals, such as body weight support or weight differentiation, is almost eliminated. Signals from proprioception of the lower body are weakened. At the same time, the otolith canal information without the gravity vector to act on the otoconia differ from that on Earth during the execution of similar movements (Kozlovskaya, 2018). In addition, signals from afferents may be distorted during adaptation to microgravity exposure, as with changes in the density of otoconia, as shown by Boyle et al. (2001), and changes in the synapses of type II hair cells identified by Ross (2000).

Locomotor disturbances are a natural consequence of SF. The noticeable instability of the gait among crew members is observed even after relatively short expeditions (from 72 h to 16 days): the gait becomes uncertain, legs are widely spaced, arms are kept apart, the body shifts from side to side at every step (Gazenko et al., 1976; Paloski et al., 1994; Reschke et al., 1998). Exposure to microgravity leads to changes in many neurological areas, including changes in perception, movement, cognition, and coordination (Moore et al., 2019). Studies carried out on weightlessness identified a wide range of changes in the muscle periphery—atony, atrophy, and decrease in power and velocity characteristics (Kozlovskaya et al., 1984; Kohn and Ritzmann, 2018). Each of these disturbances may influence the work of the motor control systems (Kozlovskaya, 1976; Edgerton et al., 2000) and be a factor in the development of disturbance of postural regulation and precise locomotor coordination (Homick et al., 1977; Paloski et al., 1994; Bloomberg and Mulavara, 2003; Melnik, et al., 2006). The brain changes associated with SF are complex because microgravity affects the brain through various mechanisms, such as brain fluid shift, and vestibular dysfunction (De la Torre, 2014; Penchenkova et al., 2019) have reported a diminished association between the vestibular nuclei and sensory/motor regions due to central adaptation which downregulates vestibular input during space flight lessening sensory discord, mitigating space motion sickness (Pechenkova et al., 2019). In other studies, using magnetic resonance imaging (MRI) after prolonged SF, signs of structural neuroplasticity and correction of neurogenesis (Demertzi et al., 2016; Hasan et al., 2018), an increase in the volume of white matter (Kramer et al., 2020) and the volume of gray matter in the sensorimotor and motor areas of the brain (Koppelmans et al., 2016) narrowing of the central furrow were reported (Kramer et al., 2020). According to the study conducted by Jillings et al. (2020a) MRI scans in cosmonauts, post-spaceflight showed chiefly changes in gray matter due to volume shifts and white matter volume expansion in the motor and coordination regions of the brain (Jillings et al., 2020a). Also, post-flight studies done by Lee et al. (2019) have demonstrated focal changes in white matter microstructure within multiple sensory regions including vestibular and proprioceptive processing. Normally the vestibular system works in congruence with the cerebellum and eyes to maintain spatial awareness. In SF, these facets are increasingly challenged when compared to the rest of the central nervous system (CNS; Van Ombergen et al., 2017a). Specifically, for the vestibular system, the functionality of the otolith organs is more affected than the semicircular canals due to their specialized role in detecting linear accelerations (Cassady et al., 2016). This creates a sort of space sickness, as the body now thinks there is much more angular acceleration than linear, which can cause nausea, vomiting, etc. just as motion sickness would. Within the first 2–3 days in SF up to 60%–80% of astronauts experience space motion sickness which can affect their operational performance (Mann et al., 2022).

Despite the above, astronauts and cosmonauts successfully perform a high volume of physical activity during SFs. It would be impossible without a profound reorganization of the sensorimotor system, the mechanisms of which have not been sufficiently studied. That requires further study human sensorimotor system in a ground-based simulation of SF exposure.

Known ground-based models have different limits on the duration of exposure and various effects on the sensorimotor system. This review aims to discuss and summarize the influence of such microgravity models as dry immersion, head-down bed rest, parabolic flight, unilateral lower limb suspension, and immobilization.

The objectives of this work are to 1) describe the technology and current possibilities of using each model, 2) describe current concepts about the effects of these models on the human sensorimotor system, and 3) define the main features of these models, determining their selection in various experiments.

This review can help actualize the information that researchers need to effectively design experiments using ground-based models of SF effects, depending on the question posed.

## 2 Dry immersion

### 2.1 Technology overview

**FIGURE 1:**
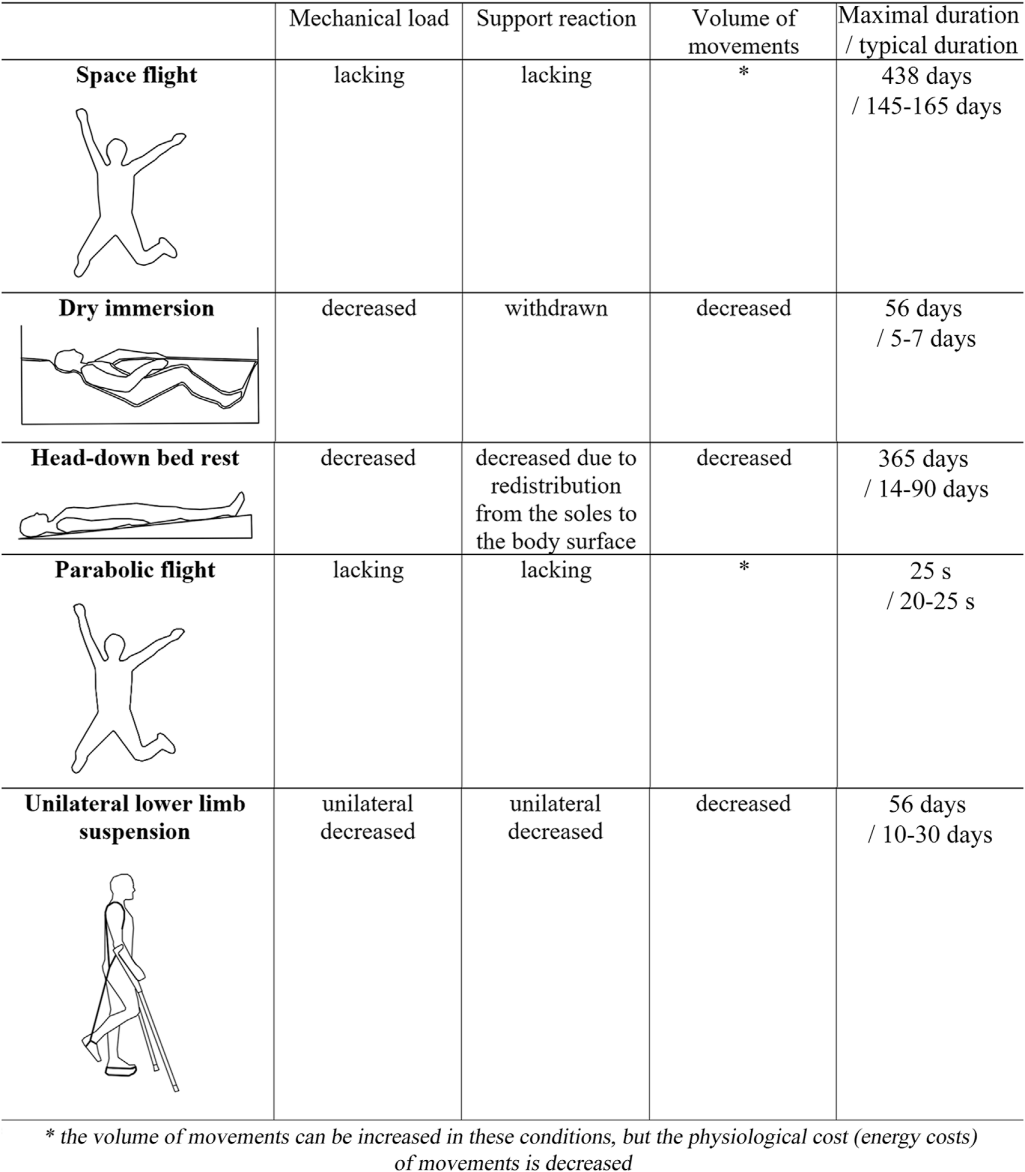
Biomechanical factors effecting the sensorimotor system under real microgravity and onground simulation.

In the early 1970s Drs. E.B. Shulzhenko and I.F. Vill-Villiams—two researchers from the Institute of Biomedical Problems (Russia)—developed the dry immersion (DI) model (Shulzhenko, 1975; Shulzhenko and Vill-Villiams, 1975). Technology and specifics are detailed in Tomilovskaya et al. (2019), and Navasiolava et al. (2011). The model suggests that the research subject is immersed in a deep bathtub with thermoneutral water (about 32°C–34.5°C) to the neck level in the supine position. The participant is separated from the water by a waterproof fabric, which surface area significantly exceeds the surface area of the water. The folds of the waterproof fabric freely envelop the subject’s body from all sides, while the bath is large enough (dimensions 2–3 × 1–3 × 1–2 m) so that the subject does not touch its walls (Figure 1).

In other words, DI is based on the anti-gravity effect of water on the human body immersed in it, which is known as Archimedes’ Principle: a body immersed in a liquid or gas is subjected to an upward buoyant force (Archimedes’ force F_a_) equal to the weight of the liquid or gas displaced by the body. The buoyant force opposes gravity, reducing the weight immersed in the water. This phenomenon makes it possible to use DI to imitate weightlessness. The internal hydrostatic pressures are exactly counterbalanced by those of the surrounding water; hydrostatic compression induces a prompt thoracocephalic fluid redistribution. Due to this, DI creates a factor of elimination of the vertical vascular gradient and the effect of the body fluid redistribution, similar to SF.

The DI can be considered one of the most widely used ground-based microgravity models (Tomilovskaya et al., 2019). Note that there are other variants of the use of water immersion in scientific research aimed at various problems. Solutions of sodium chloride or silicon may be an immersion medium (Saenko et al., 2018). Immersion can be wet—without the use of waterproof fabric (Kositsky and Avela, 2020). Immersion can also be in a standing position (Prefaut et al., 1976; Leeder et al., 2015) or sitting (Leeder et al., 2015). The temperature of the immersion medium can be cold (Sánchez-Ureña et al., 2018; Ihsan et al., 2020; Kositsky and Avela, 2020) or warm (An et al., 2019; Sugawara and Tomoto, 2021). Immersion can be for the entire body with the head (Elia et al., 2021) or a part of the body (Roberts et al., 2015; Engelland et al., 2020). It is worth mentioning the existence of the “Trout” thin-walled immersion suit equipped with an inflatable neck pillow and designed to create postural, vestibular, and operational conditions similar to SFs. Its main difference from DI is the exclusion of strict hypokinesia and maintenance of vertical posture (Genin et al., 1988).

The above–mentioned studies with immersion in cold water in various body positions are mainly aimed at studying the effects of the influence of ambient temperature on the restoration of physiological parameters after training, in hot water to search for health effects on the body during such immersion. Immersion in a medium with a higher density compared to water is used to increase the hypotensive effect of immersion in patients with high blood pressure. Studies with immersion in standing and sitting positions, as a rule, do not have the necessary weight unloading to simulate the effects of microgravity on motor control.

The wet immersion method was widely used as a microgravity model before the DI approach. The appearance of DI made it possible to use long exposures (up to 56 days), whereas when using the wet model, immersion could hardly last more than a few hours (Tomilovskaya et al., 2019). Willis showed that local water exposure at test skin sites of 4 cm^2^ cause subacute dermatitis in as little as 72 h (Willis, 1973). During whole-body immersion, skin maceration might occur faster. Nevertheless, for almost 40 years (from 1980 to 2018), DI experiments had no more than seventh days. Only in 2018–2019, a complex 21-day DI study with 10th male volunteers was conducted at the Institute of Biomedical Problems (Russia, Tomilovskaya et al., 2021). Although this long experiment allowed the researchers a deeper look into the effects of microgravity on the human body, there were some challenges, for example, hyperhydration maceration of the skin of the feet covered by the fabric. Petechial rashes in the distal parts of the lower limbs appeared during the first 2–3 min after verticalization of the subjects after 10th days of DI exposure, indicating possible changes in the microvasculature under these conditions (Tomilovskaya et al., 2021).

A landmark event for this model was the first 3-day DI experiment with the participation of six female subjects in 2020 in Russia (Nosikova I. et al., 2021; Tomilovskaya et al., 2021) since earlier DI experiments were performed only in males. Experiments involving females continue. For example, in 2021th in the Medes space clinic (Toulouse, France), 20 female subjects participated in a 5-day DI study (https://www.esa.int/ESA_Multimedia/Images/2021/09/Female_volunteer_in_dry_immersion_study), in 2022nd in the Institute of Biomedical Problems (Moscow, Russia), 16 female subjects also participated in a 5-day DI study.

### 2.2 Impact on sensorimotor system

The key factors affecting the sensorimotor system in DI are supportlessness, axial unloading, and hypodynamia (Kozlovskaya et al., 2018). Important evidence for this is the effectiveness of support stimulation (Miller et al., 2004; Kozlovskaya et al., 2007) or the axial loading suit (Rukavishnikov et al., 2017; Sosnina et al., 2020) in the prophylaxis of sensorimotor effects after DI. However, it is possible that the elimination of the vertical vascular gradient in DI can also affect human sensorimotor function (Amirova, 2018).

In keeping with the objective of describing the effects from central to peripheral, let us consider the results of a study of the rhythmic activity of the brain during DI (Lazarev et al., 2018). The power of oscillations of the alpha range (8–13 Hz) on the electroencephalogram (EEG) was shown to increase significantly on the fourth and fifth days of DI exposure, whereas significant changes in the theta (4–7 Hz), beta (14–19 Hz) and delta (1–3 Hz) ranges, as well as in the total absolute power of EEG, were not observed. The results of this study indicate that the changes in relative power were due to changes in the general pattern of neural activity and not due to a local independent increase in absolute power in the alpha range. The authors suggested that the increase in the power of alpha rhythm was not due to changes in any particular physiological system, but rather due to a decrease in afferent signals. The observed increase in alpha oscillations is also found in space flight and may be a reflection of the modulation of activity in the vestibular network due to a decrease in signals associated with gravity. Since gravity-related signals decrease, the vestibular network which includes the parietal cortex becomes inactive, which is indexed by more pronounced alpha oscillations (Cheron G et al., 2006). Also, Cebolla et al. (2016) observed the potentiation of an alpha event-related synchronization in microgravity while a low-level visuo-attentional task. The bilateral motor cortex was involved in this effect, probably responding to the high demands of continuous readjustment or maintenance of an appropriate body posture while free-floating. In addition, the SF condition was characterized by the cerebellum and the vestibular network involvement to integrate partially reduced or incongruent vestibular information (Cebolla et al., 2016). During SF, Petit et al. (2019) show a global increase in theta oscillations (5–7 Hz). Such an increase in local sleep-like episodes may be caused by increased sleep pressure (Petit et al., 2019), which is apparently not typical for DI conditions. Relatively recent studies of vestibular function in DI (Glukhikh et al., 2020; Naumov et al., 2021) confirm earlier results (Repin, 1981; Kornilova et al., 2008; 2013) about increased sensitivity to vestibular signals in the absence of direct influence of DI on the vestibular apparatus.

During DI exposure, participants are still in Earth’s gravity influence, and the vestibular system is still loaded and should yield veridical information when stimulated with head movements in any of the analog conditions on Earth. This may potentially point to the reweighting of sensory utilization post-DI in this case and or the influence of proprioception/somatosensory system on the vestibular nuclei (Jian et al., 2002; Reschke et al., 2009; Mulavara et al., 2012; 2018; English et al., 2019). At the same time, recent studies indicate a high interconnection of sensory systems with each other under conditions of support withdrawal, as well as the presence of changes in this interconnection in CNS. These studies, in particular, revealed a correlation between indicators of vestibular function and visual tracking (Glukhikh et al., 2020), an increase in the contrast sensitivity of the visual system in the low spatial frequency range with a specific sensitivity of the magnocellular pathway to these frequencies (Sosnina et al., 2019; 2021; Shoshina et al., 2020). The results of Pasekova et al. (2020) revealed a significant decrease in transient-evoked otoacoustic emissions for the stimulation frequency of 1 kHz when the subjects were in an immersion bath (Pasekova et al., 2020). Apparently, DI allows a simulation of the sensory conflict associated with a change in incoming weight-bearing sensory information. The sensory conflict is also found in SF (Jillings, 2020b; Stella et al., 2021; Tays et al., 2021; Kourtidou-Papadeli, 2022). However, during SF, it is primarily related to the conflict between otoliths and canals and may be associated with the otolith tilt-translation reinterpretation hypothesis (Reschke et al., 1984a; Merfeld, 2003). At the same time, there is a potential similar re-interpretation for the proprioception (Roll et al., 2009). It was also shown that the cortical organization of voluntary movements undergoes changes (Kirenskaya et al., 2006; Tomilovskaya et al., 2008), the systems of posture and locomotion control are severely affected (Melnik et al., 2006; Shpakov et al., 2008; Sayenko et al., 2016; Amirova et al., 2017), and the accuracy of motion control decreases (Sosnina et al., 2016). At present, little is known about the dynamic changes in the CNS caused by cerebrovascular changes that may be involved in the reorganization of the brain (Kermorgant et al., 2020), however, during a 3-day DI, no significant changes in cerebral blood flow in the cerebral arteries were detected (Ogoh et al., 2017). Note that no cerebral MRI studies of the DI model have been published so far.

It is assumed that changes in the work of the CNS under conditions of support unloading may be the cause of a decrease in inhibitory effects on spinal reflexes, however, this statement remains the subject of discussion. The phenomenon of spinal hyperreflexia is observed both after SF (Reschke et al., 1984b; Kozlovskaya et al., 1988; Grigoriev and Ushakov, 2013) and after DI (Zakirova et al., 2015; Acket et al., 2018). A recent study of the excitability of the motor neuron pool in the soleus and gastrocnemius muscles in terms of values of the thresholds and amplitudes of the motor response to transcranial and transspinal magnetic stimulation after DI confirms the spinal nature of the development of hypogravitational hyperreflexia. At the same time, the authors noted that a decrease or increase in the amplitudes of the motor response is possible, but not necessary for the state of hypogravitational hyperreflexia, while a decrease in reflex thresholds can be considered as a hallmark of this phenomenon (Nosikova I. N. et al., 2021). This phenomenon is part of pictures of “gravitational ataxia” and “muscular hypogravity detraining” syndromes, which were described earlier based on the studies performed in SF, onground models, and animal experiments (Kozlovskaya, 1983; 1988; Shenkman et al., 1997; Recktenwald et al., 1999; Ohira et al., 2002; Kakueva and Kaplanskii, 2005; Reschke et al., 2009). The removal of the support is a key in the development of these syndromes—the activity of slow muscle units reflexively decreases, causing the rapid development of atony of extensor muscles, and probably a decrease in their proprioceptors activity, leading to changes in the structure of these muscles, a decrease in their speed-strength properties, and a change in the quality of movement control (Kozlovskaya, 1983). At the same time, further work is necessary to clarify the mechanisms and significance of changes in cortical-spinal excitability in response to the unloading of the lower extremities. Roberts et al. (2010) based on the results of fourth subjects during 90-day bed rest with a tilt angle of −6 hypothesized that corticospinal excitability has a direct relationship with individual performance and may be associated with the plasticity of the cerebral cortex (Roberts et al., 2010).

It has now been confirmed that DI causes a change in the order of recruitment of soleus and gastrocnemius lateralis muscles motor units (MUs) by suppressing the recruitment of small (tonic) MUs and facilitating the recruitment of large (phase) MUs (Shigueva et al., 2015). There is an assumption that this interconnection may be the cause of a decrease in muscle tone (Kirenskaya et al., 1986), which in turn is a consequence of DI (Gevlich et al., 1983; Miller et al., 2010; Ogneva et al., 2011). While early studies of muscle tone, mainly by assessing the transverse stiffness of the muscles, demonstrated its significant decrease in the extensor muscles of the lower leg (in the gastrocnemius and soleus) and the absence of its changes in the lower leg flexor (tibialis anterior), but the most recent data using other measurement methods indicate that this statement is not so unambiguous. In the acute period of adaptation to DI, in the first 2 h of exposure, there was a significant decrease in the tone of the tibialis anterior and soleus muscles accompanied by an increase in the tone of the gastrocnemius muscle (Amirova L. E. et al., 2021; Amirova et al., 2021 L.). Similar results were found in the study of transverse stiffness and tone of the longissimus dorsae muscle in 3-day and 6-hour DI. It is worth noting that a pronounced decrease in stiffness correlated with an increase in the severity of back pain during DI, while no similar correlations were found with changes in muscle tone. The authors suggested that muscle tone and back pain are determined by a large number of intermediate factors (Rukavishnikov et al., 2017). Moreover, a study of muscle tone in a long-term 21-day DI showed a decrease in the tibialis anterior muscle and an increase in the soleus and gastrocnemius muscles, which may be the result of functional compensation through increasing fascial contractility (Amirova et al., 2020). A similar phenomenon was also observed in a study of the dynamics of the morphological and functional characteristics of the foot structure in a 5-day DI (Saveko et al., 2021), as well as in the rectus femoris muscle in a 3-day DI (Demangel et al., 2017). Furthermore, Reschke et al. (2009) noted a decrease in the stretch reflex parameters of the postural muscles after exposure to long-duration head-down bed rest: a decrease in start latency and peak latency of the monosynaptic stretch reflex, peak magnitude and latency of the functional stretch reflex (Reschke et al., 2009). Due to the above, the questions on the effect of DI on muscle tone and the mechanisms of this effect are open for further research and discussion.

Morphological studies of muscle tissue made another contribution to understanding the mechanisms of DI and linking those to the effects during SF. The elimination of support afferentation was shown to inactivate the pool of slow motor units, which leads to selective inactivation and subsequent atony and atrophy of muscle fibers (Shenkman and Kozlovskaya, 2019). Fibers that lost a significant part of the cytoskeleton molecules are not capable of effective actomyosin motor mobilization, which results in a reduction in sensitivity to calcium and a decrease in the range of maximum tension of permeabilized fibers. Support unloading in DI also leads to a reduction in the effectiveness of protective mechanisms (such as nitric oxide synthases), a decrease in the activity of AMP-activated protein kinase, a decrease in the relative content of titin, nebulin, desmin, and α-actinin (Shenkman and Kozlovskaya, 2019; Sharlo et al., 2021). A comprehensive study of acute changes during 3-day DI in the morphological parameters of muscles and speed-strength characteristics of the maximum isometric voluntary contraction showed the relationship between these phenomena (Demangel et al., 2017). Demangel et al. (2017) also found an increase in the amount of NCAM-positive myofibrils, which indicates an increased percentage of denervated fibers and early changes in MU recruitment models. A decrease in the speed-strength properties of muscles was also observed earlier (Koryak, 1998), and the authors also associated this phenomenon with a decrease in the intensity of the central efferent command.

## 3 Head-down bed rest

### 3.1 Technology overview

Since the 1960s, the head-down (with an angle of −6–8) bed rest (HDBR) was the major ground-based model of the effects of weightlessness. Technology and specifics are detailed in Pavy-Le Traon et al. (2007); Fortney et al. (1996). The key role in the development of one of the first HDBR experiments played by Russian cardiologist Evgeniy Ivanovich Chazov (Grigoriev et al., 2018). These experiments allowed researchers to obtain data on changes in the physiological systems of the body and test new means and methods to prevent adverse consequences of SF. The HDBR model became more widespread in the early 1970s. In 1972, Voskresensky et al. (1972) published the first data that the anti-orthostatic position with an inclination angle of −4 is a more adequate model of the effects of weightlessness than the usual horizontal position with limited mobility (Voskresenskiy et al., 1972). Finding the most adequate tilt angle during bed rest was an important objective for researchers (Mikhaĭ;lov, 1979). Even now, used angles in HDBR vary from −4 to −15, depending on the aims of the study. The most common angle for modeling the effects of SF is currently −6, and many researchers recognize such conditions as the international standard for weightlessness modeling (Smith et al., 2011; Pandiarajan and Hargens, 2020), however, this question is still debating.

During the HDBR, the body weight is not eliminated, but rather the gravity vector is transferred from the head-legs to the chest-back direction. At a −6 bed rest with the head tilted down, the G_z_ vector is practically leveled, so that the body adapts to the G_x_ vector from front to back in the supine position (Figure 1). A tilt angle of −6 gives approximately −0.1 G_z_ [ = sin (−6)] (Watenpaugh, 2016). In this position, headward fluidshift occurs; bones, most muscles and the heart work much less against gravity (G_z_) compared to their normal function on Earth.

Compliance with the standards for conducting research using the HDBR model means that any exercise, showering, and toileting should be performed in a perfectly horizontal or anti-orthostatic posture to ensure the best accuracy of microgravity simulation in space (Hargens and Vico, 2016). This is also the case during DI. Often, in order to reduce health risks during eating, a short-term change in the position of the subject’s body is allowed. For example, while in DI a pillow is placed under the upper body of the participants, during HDBR, the participants are allowed to lean on an elbow or use a pillow to take a comfortable eating position. In HDBR, leaving the anti-orthostatic posture even for a short time, as well as the use of a pillow to support the head, were shown to significantly affect intracranial pressure and can neutralize possible symptoms of visual disorders (Lawley et al., 2017).

Both male and female subjects participated in HDBR since the 1980s (Convertino et al., 1977; Pavy Le Traon et al., 2007). An important distinguishing feature of the use of HDBR is the possibility of a long duration of exposure. For example, as early as 1979, such an experiment lasting 182 days was reported (Bychkov et al., 1979), which is more than 3 times longer than the duration of the longest experiment with DI. The longest-ever experiment with HDBR (angle of inclination of 4.5) lasted 370 days (1987–1988) and was performed on 10 men, five of whom used means of countermeasure to the effects of microgravity (Grigoriev et al., 2018). Note that a lower number of study participants was mistakenly reported by Hargens and Vico 2016.

### 3.2 Impact on sensorimotor system

The HDBR model reproduces most correctly the following factors of SF: reduction in static and dynamic muscle loads, redistribution of the body fluids in the cranial direction, and removal of the support load from the receptor surface of the feet (Grigoriev et al., 2018).

Functional magnetic resonance imaging (fMRI) allows researchers to study patterns of central activity and connectivity during HDBR. The findings from studies using fMRI in HDBR demonstrated a reduction in connectivity at rest in the thalamus after third days of HDBR (Liao et al., 2012), in the anterior insula and anterior middle cingulate cortex after 45 days of HDBR (Zhou et al., 2014), in the motor, somatosensory, and vestibular areas of the brain after 70 days of HDBR (Cassady et al., 2016), as well as a widespread increase in gray matter in the posterior parietal areas and its decrease in the frontal areas (Koppelmans et al., 2017). These results are believed to be primarily associated with a decrease in motor activity (Stella et al., 2021), however, it is possible that in this case the leading role in the identified changes is played by a decrease in the gravity reference afferent inflow. A similar trend toward an increase in gray matter tissue in the basal ganglia was observed in SFs, which indicates adaptive sensorimotor neuroplasticity (Jillings et al., 2020a; Doroshin et al., 2022). Also, fMRI studies of brain activity after a long SF revealed a decrease in connectivity at rest in the right insula, between the left cerebellum and the right motor cortex. At the same time, a decrease in connectivity at rest after SF in the right insula which is part of the vestibular cortex indicates changes in the integration of neurosensory information: vestibular, visual, and proprioceptive (Demertzi et al., 2016). In a study by McGregor et al. (2021a) during 30 days of HDBR + CO_2_, five out of 11 subjects showed a sign of spaceflight-associated neuro-ocular syndrome (SANS)—optic disc edema, that was accompanied by a slowdown in reaction and movement time after exposure when performing a visuomotor adaptation task using a joystick (Banker et al., 2021) and greater visual contributions to balance (Hupfeld et al., 2020). Interestingly, in six subjects without signs of SANS (NoSANS), there was a greater decoupling between the insula and the other regions within the network during the baseline phase, as well as a decrease in connectivity between the right posterior parietal cortex and clusters in bilateral insular cortices during HDBR + CO_2_ (McGregor et al., 2021a). These observations are consistent with the results of Pechenkova et al. (2019), namely, indicating that such a decrease in connectivity may be associated with the theory of central adaptation, which downregulates vestibular input during space flight lessening sensory dissonance. Functional connectivity decreases involving the insula and posterior cingulate cortex at rest are the most notable commonalities between HDBR and SF (McGregor et al., 2021b). After 70 days of HDBR, brain activity in the cerebellum and visual areas was significantly increased with foot movement. The degree of this increase positively correlated with indicators of functional mobility and balance control, which indicates not only adaptive changes in neural control during prolonged bed rest but also a close relationship between changes in the activity of the CNS and locomotor and postural functions (Yuan et al., 2018a). For example, disturbances in static and dynamic postural characteristics were previously noted after HDBR (Reschke et al., 2009; Viguier et al., 2009). Furthermore, a greater increase of activation in multiple frontal, parietal, and occipital regions in response to vestibular stimulation during HDBR was associated with greater decrements in balance and mobility from before to after HDBR, suggesting reduced neural efficiency (Yuan et al., 2018b). Although the results of EEG in HDBR confirmed the increase in alpha power observed during actual SF, contrary results were also reported (Han et al., 2001). Since the gravitational stimulus is preserved in HDBR, some authors believe that it is not the best model for studying the impact of microgravity on the human brain (Marušič et al., 2014; Van Ombergen et al., 2017a).

The study of the features of the rhythmic activity of motor units before and during a 120-day HDBR demonstrates the advantage of this model from the point of view of the duration of exposure. For example, during the first 14–30 days of HDBR, a sharp increase in the variability of interpulse intervals and an increase in the degree of synchronization of motor unit activity were observed; starting from the 30th day of HDBR, a reproducible decrease in the duration of interpulse intervals was observed, as well as the disappearance of synchronization of motor units while maintaining a high level of variability in spike activity. The obtained results allowed the authors of this work to separate the effects of HDBR exposure into two stages: at stage 1, the changes were associated with reflex reactions to support unloading, at stage 2, with the process of postural muscles atrophy (Kozlovskaya and Kirenskaya, 2004). The phenomenon of hypogravitational hyperreflexia occurs in HDBR as well as in DI and after SF (Kozlovskaya et al., 1988; Saenko et al., 2000; Roberts et al., 2010), however, despite this and other similarities in the impact on the human sensorimotor system, a comparison of the effects of DI and HDBR shows the superiority of DI in the dynamics and depth of these changes (Aleksandrova et al., 1980; Chaika et al., 1982; Kozlovskaya et al., 1984; Grigoriev et al., 2004; Tomilovskaya et al., 2019). After 20 days of 6 HDBR, the value of the maximum H-reflex (Hmax) of the soleus muscle, expressed as a percentage of the maximum M-response (Mmax), decreased from 36.6% (Yamanaka et al., 1999), when a similar parameter on the seventh day of DI decreased by 20% (Zakirova et al., 2015). Electroneurographic findings have suggested decreased tibial nerve M-responses after second and fourth mouth of HDBR (Ruegg et al., 2003), similar to DI (Zakirova et al., 2015). After 5 days of DI, the maximum amplitude of the motor evoked potential of the soleus muscle while transcranial magnetic stimulation was almost twice the baseline values (Nosikova I. N. et al., 2021). At the same time, in a study by Yamanaka et al. (1999) and Miyazaki et al. (2002) there were no significant differences between the average amplitudes of the motor evoked potential of the same muscle before and after HDBR.

A comparison of the transverse stiffness of the tibialis anterior and soleus muscles also demonstrates a more rapid development of changes in DI (Gevlich, 1984). The transverse stiffness of the soleus muscle decreased by 15% after 7 days of SF, by 16% after 14 days of 4 HDBR, by 29% after 7 days of DI, and by 47% after 7 days of combined DI (daytime) and 4 HDBR (nighttime; adapted in Amirova L. et al., 2021). A study of a similar parameter by Kozlovskaya et al. (1988) showed that on day 3 of DI and day 31 of HDBR, there was a comparable decrease in the stiffness of the soleus muscle.

The amplitude of maximal voluntary muscle contraction (MVC) of the lower leg was equally decreased after 7 days of SF, 7 days of DI, and 4 months of HDBR (Koryak, 1998; Koryak, 2001). Demangel et al. (2017) registered a decrease of about 11% in the quadriceps MVC during a 3-day DI, while a 15% reduction in the quadriceps MVC was observed only after 5 weeks of HDBR (Krainski et al., 2014). Also, according to Shenkman et al. (1997), the decrease in the size of slow and fast types of muscle fibers by the seventh day of DI reached 15%–18%, whereas, in HDBR, similar changes were observed only after 40–60 days of exposure (Berg et al., 1997). Overall, recent studies of cardiovascular, postural, and neuromuscular changes during 21-day HDBR and 3-day DI found similarities in their effects, suggesting that DI is an accelerated model compared to HDBR (Tomilovskaya et al., 2018). However, it was the use of the HDBR model that made it possible to confirm under strictly controlled laboratory conditions the specific features of the order of transformation of muscle fibers identified by Fitts et al. (2010) during a long 6-month SF: type I soleus muscle > type II soleus muscle > type I gastrocnemius muscle > type II gastrocnemius muscle (Trappe et al., 2004; Krainski et al., 2014; Hargens and Vico, 2016). It should be noted that a 60-day HDBR led to a decrease in the cross-sectional area (CSA) of the muscles of the lower back by 10% (Holt et al., 2016), a short-term (17–20 days) HDBR—a decrease in the CSA of the lower leg muscles by 10%–12% (Akima et al., 2001), and a 90-day HDBR—a decrease in the CSA of the lower leg muscles by 26% (Rittweger et al., 2005). At the same time, the atrophy correlated with a decrease in the contraction force of these muscles (Koryak, 2014). After 84 days of HDBR, MRI showed a 17% decrease in weight and a 40% decrease in the strength of the lateral thigh muscle (Trappe et al., 2004). It was also shown that not all muscles of the lower limbs are affected by long-term HDBR: after 60 days of HDBR, there was no significant decrease in the volume of the rectus femoris, adductor brevis, gracilis or pectineus muscles compared with the baseline level (Akima et al., 2001).

## 4 Parabolic flight

### 4.1 Technology overview

Flying a parabolic trajectory in an airplane, or parabolic flight (PF), is a unique way to create free fall on Earth, which is important for astronaut training and scientific research. The technology and specifics of PF are reviewed in detail by Karmali and Shelhamer (2008). Note that the authors emphasize that it is important to distinguish between a “free fall” and “weightlessness”. For example, in an orbital flight, a spacecraft in a near-Earth orbit and its crew are constantly falling toward the Earth under the influence of the Earth’s gravity, but they have the sufficient tangential speed to keep them at the same distance from the Earth. That is, the sum of velocities toward and parallel to earth keeps spacecraft at the same distance from earth, in constant free fall, but gravity is not equal to zero. In this case, weightlessness is a subjective feeling for humans, because, according to Einstein’s principle of equivalence, no simple physical sensor can determine whether the applied acceleration is due to gravitational or inertial force, including sensory organs in the human body. By a similar principle, the PF creates a free fall, following a trajectory in which the acceleration of the aircraft cancels for the acceleration due to gravity along the vertical axis of the aircraft. Thus, if the aircraft and its passengers “fall” together with an acceleration of 9.81 m/s^2^, they find themselves at 0 g, so that the aircraft is not acting on the passengers. Such a flight usually consists of 15–60 parabolas, each of which provides about 20–25 s of free fall. Between parabolas, the aircraft must climb again, typically within a 40 s interval. In this process, the speed of descent of the aircraft decreases and eventually becomes the speed of ascent, and the acceleration levels reach 1.5–1.8 g. The entire flight usually continues for 3–3.5 h. Thus, it is worth noting that the actual level of gravity during PF along the vertical axis of the aircraft demonstrates a longer action of hypergravity on the human body than microgravity. Moreover, the pitch rotation of an aircraft (3°/s on average) is almost undetectable by the vestibular system but may influence some physiological parameters (Jones and Milsum, 1971). Researchers need to consider these details when designing, analyzing, and interpreting experiments under conditions of PF (Karmali and Shelhamer, 2008). White et al. (2020) also noted that alternating between 1 g, 1.8 g, and 0 g may affect adaptation measured in PF. Finally, the numbers of participants and experiments per parabolic flight campaign are rather low. Typically, one campaign consists of three PFs with two to three participants in each flight, which results in six to nine participants for a single study. Operational and budgetary constraints often make it difficult to run the same experiment over several campaigns, which sometimes makes data collection difficult when there is a large number of participants.

PF also makes it possible to reproduce gravity conditions on Mars (0.38 g) for 32 s and on the Moon (0.16 g) for 25 s (Pletser et al., 2012), but this review only considers the results obtained by modeling 0 G.

### 4.2 Impact on sensorimotor system

Among the factors affecting the human body in PF, a consistent and rapid change between normo-, hyper- and microgravity can be distinguished. It can be assumed that the studies of the sensorimotor system carried out before and after the end of the entire PF rather reflect the response to gravitational transitions. However, measurements taken directly within the 20–25 s interval of supportlessness at 0 g reflect the short-term acute effects of such factors as axial and support unloading, elimination of the vertical vascular gradient, unloading of the otolith organs, not excluding the possible effect of the previous 20 s hypergravity exposure.

Data from fMRI scans before and after PF with the participation of 28 volunteers revealed decreased intrinsic connectivity at the right temporoparietal junction (rTPJ), an area involved in multisensory integration and spatial tasks (Fiori et al., 2015), as well as a decreased in intra- and interhemispheric anticorrelations between rTPJ and supramarginal gyri, which indicates both altered vestibular and intrinsic functions. The authors noted that the observed changes demonstrate how the brain copes with gravitational transitions (Van Ombergen et al., 2017b); it is also likely that they are associated with the process of suppression of conflicting vestibular signals (Van Ombergen et al., 2017a). Interesting data were obtained by (Schneider et al., 2008; Schneider et al., 2009) when recording EEG activity during PF, as well as under normal gravity in various body positions (lying on the back, sitting, and with the head tilted down by 9). Beta-2 frequency activity was increased in the right superior frontal gyrus in the normal gravity phase of the flight. The power of alpha-2 frequencies was significantly reduced in the hypergravity phase compared to the normal gravity phase. The most notable aspect of these data is the suppression of increased frontal beta-2 activity during the microgravity phase. Notably, the data do not reflect the effects of continuous exposure to microgravity. Rather, the data reflect the recurring and instantaneous effects of altered gravitational forces. Since experiments with HDBR showed changes in the left inferior temporal gyrus in the supine and tilted positions, the authors concluded that the observed changes in weightlessness conditions cannot be explained by hemodynamic changes, but rather reflect the emotional processes associated with the experience of weightlessness. This assumption can also be supported by the noted increase in beta oscillations from the preflight to flight phase (Schneider et al., 2008; 2007). However, there is an opinion that such changes may be the result of baroreceptor stimulation (Lipnicki, 2009) or a decrease in excitation levels (Wiedemann et al., 2011). It is important to emphasize that under conditions of SF the alpha rhythm increases in the parietal-occipital and sensorimotor areas, which is believed to be associated with an elimination of the action of gravity in space (Cheron et al., 2006; Van Ombergen et al., 2017a).

An important aspect is a fact that parabolic flight causes a symptom complex of space motion sickness, indicating the presence of a sensory conflict under the influence of PF factors (Kornilova and Kozlovskaya, 2003) and the feasibility of using this model to investigate preventive measures for this condition (Russomano et al., 2019).

Analysis of the PF data on the dynamics of various parameters reflecting the state of the sensorimotor system demonstrates how this model can be used for an immediate comparative assessment of the influence of various gravitational levels on the human body. For example, the illusion of arm movement created by muscle vibration increased during the 1.8 g phase and decreased during the free fall phase (Lackner and DiZio, 1992). The researchers concluded that gravity affects the power of muscle spindle receptors per unit of spindle stretch in the following way: otolith unloading in microgravity conditions reduces the downward modulation of the α- and γ-motor neurons, which leads to a decrease in tonic vibration reflexes. Alternatively, increased gravitational load on the arm could be equivalent to resistance (Lackner and DiZio, 1992). Testing of motor coordination of the hands (aiming and tapping with pencil and paper) in both normal and alternating gravity in PF showed that changes in the g level cause errors in the direction of the gravity axis - the hand aims too high at 0 g and too low at 2 g. Interestingly, there were more errors at 0 g than at 2 g. In addition, there was a downward trend in velocity at 0 g for all motion orientations (Ross, 1991). A change in the motor strategy of the hand during g-transitions was also observed in the study by Boulanger et al. (2021). It is interesting that elastic bands on the arm, simulating the gravitational torque in the shoulder joint, helped to significantly increase the accuracy of arm movements during PF, bringing it closer to the ground level. The authors concluded that the decrease in the proprioceptive function of the forearm in weightlessness conditions is a consequence of the absence of torques in the joints (Saradjian et al., 2013; Weber and Proske, 2022). Macaluso et al. (2017) reported a change in the kinematic strategy of body movements in PF at 0 g and its similarity with the strategy observed in the prototype of a spacesuit simulating immersion in water (AquaS environment; Macaluso et al., 2016), namely, there was some “simplification” of the postural control—a reduction in the number of degrees of freedom, which helps to minimize the cost of mechanical energy and maximize the smoothness of the joints (Gaveau et al., 2014; Hilt et al., 2016). When walking and running in the 0 g PF phase, there was an increase in hip flexion, an increase in the amplitude of motion in the hip joint, an increase in the duration of contact with the support surface (De Witt et al., 2010), as well as a redistribution of the support load on the forefoot (McCrory et al., 1997), similar to the walking strategy in space flight conditions (Saveko et al., 2020). It was also shown that periods of hypergravity in PF can affect the perception of one’s own body movements. Even if during the period of hypergravity the apparent stability of the self and the environment was gradually restored, then with repeated exposure, voluntary locomotion again was causing an illusion of the motion of the self and the environment (Lackner, 2018). At the same time, in PF, similar to SF, there was an increase in the value of the visual afferent inflow to maintain equilibrium (Clement and André-Deshays, 1987). It was shown that changing the level of gravity affects the control of the isometric force of the arms: compared to the normal level of g, the forces produced were higher in hypergravity and even higher in microgravity. Vibration reduced the magnitude of the generated forces regardless of the level of g (Mierau et al., 2008). The data obtained in PF highlight the ability of the CNS to perform very fast neuromuscular adjustments adapted to gravitational transitions (Gambelli et al., 2016; Bringoux et al., 2020; Waldvogel et al., 2021).

The study of the influence of the gravity level on the excitability of the soleus motoneuron pool to the afferent input Ia while maintaining a vertical position also demonstrates the immediate development of PF effects in humans. For instance, the electromyographic (EMG) activity of the soleus muscle was the highest in hypergravity, while it was practically absent in microgravity. During normo- and hypergravity, a linear relationship between the EMG activity and the amplitude of the H-reflex of this muscle was observed. However, under microgravity, despite the almost absent EMG activity, the amplitude of the H-reflex was greater than under terrestrial gravity. Moreover, when the subjects were voluntarily contracting the soleus muscle, applying a load to the joints of the lower limbs and the spine by pulling the handle up, this increase in the H-reflex was almost disappearing. These results suggest that somatosensory systems that determine the load on the lower limbs and/or the spine may play a role in reducing the excitability of the soleus motor neuron pool to afferent inputs Ia *via* presynaptic inhibition (Miyoshi et al., 2003). Note that an increase in the amplitude of the H-reflex was also observed under the action of DI (Zakirova et al., 2015). Nomura et al. (2001) also reported an increase in the H-wave of the Hoffmann reflex of the soleus muscle at 0 g in PF. At the same time, hypergravity of 1.5 or 2 g did not affect the amplitude of the H-wave. Changes in the level of gravity did not affect the time interval between stimulation and the M- or H-wave and the amplitude of the M-wave of the soleus muscle. Although the vertical position of a person during PF was maintained due to pronounced tonic activity of the soleus muscle at 1 g and 2 g, the same posture at 0 g was maintained mainly due to the activity of the tibial muscle (Clement and André-Deshays, 1987). At the moment, there is very little information about morphological changes in human muscle tissue after exposure to PF.

## 5 Suspension

### 5.1 Technology overview

Technology and specifics of ULLS are detailed in Hackney and Ploutz-Snyder (2012). In 1991, Hans Berg et al. in collaboration with the Kennedy Space Center published the first study using unilateral lower limb suspension (ULLS) as a human model for studying the effects of unloading on skeletal muscles (Berg et al., 1991; Dudley et al., 1992a). ULLS requires the participant to perform all activities with underarm crutches while wearing a single boot with a thick sole. The raised boot eliminates ground contact with the adjacent foot, thereby unloading the lower limb. There are two main versions of ULLS in the published literature. The original model developed by Berg et al., requires wearing belts around the waist and a strap attached to a modified shoe to suspend one lower limb. The strap holds the knee in a flexed state (∼90–120), while a 50 mm platform shoe is put on the opposite foot to prevent involuntary weight transfer. However, this method was associated with a higher risk (∼2.7%) of deep vein thrombosis (Berg and Tesch, 1996; Bleeker et al., 2004). In the modified model, the strap was removed and the platform shoe was raised (by approximately 10 cm) to allow the unloaded foot to swing freely (Ploutz-Snyder et al., 1995). Elastic compression stockings were also used to prevent the accumulation of blood in the lower leg (Byrne, 2001). Most studies today use a modified ULLS model (Hackney and Ploutz-Snyder, 2012; Figure 1). ULLS is a well-known ground-based analog of microgravity; it is also one of the most cost-effective methods to study the effects of unloading on human skeletal muscles (Pandiarajan and Hargens, 2020). Increased mobility is a decisive factor in determining cost-effectiveness and patient compliance with the treatment regimen since mobility allows the subjects to travel, work, and stay at home. This reduces the costs associated with methods that require a hospital stay or constant monitoring. At the same time, reduced monitoring means that the adherence to the study protocol cannot be fully controlled, but the reduced impact on the subject’s daily life allows to recruit of more volunteers (Tesch et al., 2016). In addition to daily interviews with participants, monitoring of compliance with the experimental conditions can be carried out by measuring the temperature of the skin surface and the circumference of the lower leg. As a rule, skin temperature in an unloaded limb is approximately 2°C lower than in a loaded one, and calf circumference is approximately 2–3 cm greater (when compression stockings are not worn) in an unloaded calf compared to a loaded one (Adams et al., 1994; Tesch et al., 2004). In addition, specially designed plantar accelerometers can be used for more precise control (Cook et al., 2006). Typically, the duration of such exposure ranges from 12 h to 56 days (Campbell et al., 2019).

### 5.2 Impact on sensorimotor system

The main factors in ULLS that affect the human body are a unilateral decrease in the motor activity of the lower limb, the elimination of support and proprioceptive afferent signals, and the removal of axial load on the lower limb. It is important to emphasize that this model is difficult to compare with the study of the impact of microgravity and space flight on the human body (Pandiarajan and Hargens, 2020) since the impact in ULLS is very local. In this connection, it is more correct to consider this model as a method for studying the effect of unloading on human skeletal muscles (Hackney and Ploutz-Snyder, 2012).

To date, the authors of this review have not been able to find any data on the study of brain functions under the influence of ULLS. However, it can be assumed that even a unilateral effect of the above factors can affect the CNS, for example, by changing the perception of the body scheme, movement stereotype, etc. For instance, ULLS was noted to alter the dynamic movements of the entire body (Tesch, et al., 2016). After 3 weeks of ULLS, the ability to vertical jump both on one leg (previously unloaded) and on two legs was worsening (Horstman et al., 2012).

Quite often in the literature, there is a comparison of the effects of ULLS with the effects of bed rest without tilt (Tesch, et al., 2016). In general, it can be said that the effects of ULLS on the structural and functional characteristics of the lower limb do indeed have similar trends with the effects of the earlier considered models. After 21–28 days of ULLS, the amplitude of the H-reflex of the soleus muscle of the unloaded leg at rest increased by approximately 18%–35% (Clark et al., 2006; Seynnes et al., 2008; 2010); similar changes were observed after 35 days of bed rest (Duchateau, 1995), indicating an increase in spinal excitability.

Despite these changes, the vast majority of studies showed that ULLS does not affect the voluntary activation of motor units (De Boer et al., 2007; Seynnes et al., 2010; Horstman et al., 2012; Campbell et al., 2013; Cook et al., 2014), but there is recent evidence of motor unit dysregulation as a key component of functional loss after ULLS (Inns et al., 2022). The results of surface EMG recording showed that during the reproduction of the MVC, the EMG amplitude was decreasing compared to that before ULLS (Dudley et al., 1992b; Seynnes et al., 2008), however, when performing submaximal tasks, the EMG signal after ULLS was increasing (Berg and Tesch, 1996; Schulze et al., 2002; Tesch et al., 2004).

In two ULLS studies, the unloaded leg showed increased contrast shifts in T2-weighted MRI scans of skeletal muscles during various physical activities, indicating an increase in required muscle mass and/or higher metabolic requirements to perform a concentric muscular action (Ploutz-Snyder et al., 1995; Akima et al., 2009). These data are likely to indicate that more type IIa or type IIx explosive motor units are recruited to achieve the same level of force output after unloading (Hackney and Ploutz-Snyder, 2012). A reduction in muscle size depending on the duration (up to 42 days) of unloading the knee extensors and plantar flexors was shown using MRI or computed tomography. In total, the observed rates of reduction in the sizes of extensor knee and plantar flexor muscles (anatomical cross-sectional area or volume) per each day of ULLS comprised ∼0.40 and ∼0.36%, respectively (Adams et al., 1994; Tesch et al., 2004; Clark et al., 2006; De Boer et al., 2007; Akima et al., 2009; Seynnes et al., 2010). A summary analysis of the ULLS results showed that during unloading the vastus lateralis muscle is most prone to atrophy, followed by the gastrocnemius muscle (Hackney and Ploutz-Snyder, 2012). According to the literature, however, the longer the duration of unloading (more than 50 days), the greater the atrophy of the plantar flexors compared to the knee extensors (Narici and de Boer, 2011). Several studies showed significant fiber atrophy in combination with a marked decrease in phosphorylation of the mechanosensitive FAK protein (Flück et al., 2014), an increase in the content of 3-methylhistidine (Tesch et al., 2008), a marker of myofibril destruction (Sharlo et al., 2021), and an increase in the MuRF1 and MAFbx mRNA levels (Abadi et al., 2009; Gustafsson et al., 2010) within 3 days after the completion of ULLS. Moreover, the early activation of proteolysis was also indicated by increased expression of atrogin-1 and MuRF-1 (Flück et al., 2014). These signs of early activation of muscle protein disintegration appear to precede a marked reduction in muscle protein synthesis (by ∼50%) during a 10-day ULLS (De Boer et al., 2007).

A consequence of changes in the neuromuscular apparatus is a decrease in its functional parameters during ULLS. After 28 days of ULLS, MVC values decreased by 32.6% (Sinha et al., 2020), and strength stability assessed at moderate intensity (∼25% of MVC) decreased by approximately 22% and 12% in knee extensors and plantar flexors, respectively (Clark et al., 2007). In the knee extensors, overall isokinetic performance decreased by about 13% after 21 days of ULLS (Schulze et al., 2002), while dynamic endurance decreased by about 24% by day 30 (Cook et al., 2010). Studies of individual muscle fibers before and after ULLS show a decrease in their diameter (Malis et al., 2019) and contraction force (Brocca et al., 2015). This, together with changes in the neural and elastic components of the muscles, results in a marked decrease in knee extensor and plantar flexor muscle strength by 4%–5% a week (Tesch et al., 2016). It is important to note that the ability to produce force per unit of time also deteriorates after ULLS, and a significant decrease in the rate of strength development was reported (Clark et al., 2006), which was explained by a decrease in maximum strength rather than a change in the contractile properties of the muscles (Horstman et al., 2012).

## 6 Other methods/models

Plaster casts for the immobilization of joints can also be used to study the effects of a local radical decrease in motor activity, the elimination of support and proprioceptive afferent signals, and the removal of axial load (Sargeant et al., 1977; Gibson et al., 1987). However, the fixation of joints at different angles was shown to affect muscle tone and cellular metabolism (Booth, 1977; Goldspink et al., 1986). In contrast, human exposure to microgravity during space flight unloaded both muscles and bones, while the joints were free to move in a wide range of motion (Hackney and Ploutz-Snyder, 2012). In a study by Suetta et al. (2012), a 10% atrophy of muscle fibers after only 4 days of immobilization with a cast was reported. Even a 5-day immobilization of the knee was causing a decrease in the cross-sectional area of the quadriceps femoris muscle by 3.5% and muscle strength by 9%. At the same time, similarly to a 3-day ULLS, the expression of MAFbx and MuRF1 mRNAs was increasing (Dirks et al., 2014; Sharlo et al., 2021); however, in a 14-day immobilization of the legs, an increased content of MAFbx mRNA, but not MuRF1 mRNA, was observed in the vastus lateralis muscle of the thigh. In addition, the isometric force of the knee extensor muscles was decreasing by 27% compared with the initial level (Jones et al., 2004). Note that although the short-radius centrifuge is not a microgravity model, it can be used to imitate the symptoms of motion sickness (Lewkowicz, 2019; Frett et al., 2020). Moreover, rotation modes can be adjusted in such a way as to simulate the effects of parabolic flight. At the same time, the participants experienced an illusion of microgravity, visual-motor coordination similar to the conditions of microgravity, and a training effect that facilitated the further experience of the parabolic flight (Kowalczuk et al., 2018). The short-radius centrifuge can be used as a countermeasure in combination with the microgravity models—HDBR and DI (Clément et al., 2016). Recent data also show that isolation experiments may be responsible for the formation of a specific stereotype of movements due to the long stays of crew members in a limited space of the space station model (Saveko et al., 2022).

## 7 Methodological considerations

Having a variety of advantages and disadvantages, all of the listed approaches to modeling the effects of SF on the sensorimotor system are actively used by scientists throughout the world. Under the circumstances, the authors find it important to match the research objectives with the choice of the model of SF effects. The authors listed some main features of the discussed models in Figure 1, however, to choose the right ground-based experimental model of the SF effects, a broader approach that takes into account previously obtained results should be applied.

Understanding the role of gravity in movement control, in general, requires consideration of data from different environments and contexts. The complexity of these processes and the potential distorting factors associated with them make it sometimes difficult to interpret the results. However, the great variety of the results obtained in various ground-based models provides insight into the neural basis of gravity-dependent aspects of perceptual and motor tasks (White et al., 2020). SF-induced changes in electrocortical activity should also be considered in the context of emotional stressors. For instance, parabolic and space flights are associated with increased levels of anxiety; on the other hand, DI and HDBR can trigger boredom due to monotony and immobilization (Marušič et al., 2014), while allowing to explore of the mechanisms of sensorimotor disorders under more controlled conditions (Bock, 1998). Of interest is the comparison of data from similar measurement methods obtained in different ground-based models. For example, after 5 weeks of HDBR (Krainski et al., 2014) and after 10 days of bed rest (Kortebein et al., 2007; 2008), the degree of MVC reduction in the quadriceps muscle in men was approximately 15%, while to reduce this indicator by 11% under conditions of DI or strict immobilization, 3 days were sufficient (Demangel et al., 2017). This comparison indicates the importance of the complete elimination of support afferentation for the development of this phenomenon, as well as an increase in the rate of its development at the conditions of severe limitation of movements and old age, expanding our understanding of the mechanisms of reducing this indicator.
