# Corrigendum: Impact of different ground-based microgravity models on human sensorimotor system

**DOI:** 10.3389/fphys.2023.1281392

**Published:** 2023-08-30

**Authors:** Alina Saveko, Maria Bekreneva, Ivan Ponomarev, Inna Zelenskaya, Alexandra Riabova, Tatiana Shigueva, Vladimir Kitov, Nelly Abu Sheli, Inna Nosikova, Ilya Rukavishnikov, Dimitry Sayenko, Elena Tomilovskaya

**Affiliations:** Russian Federation State Scientific Center—Institute of Biomedical Problems of the Russian Academy of Sciences, Moscow, Russia

**Keywords:** groundbased observations, dry immersion (DI), bed rest, parabolic flight, space flight, immobilization, anti-orthostatic hypokinesia, suspension

In the published article, there was an error regarding the **Affiliation** for Dimitry Sayenko. He should only have **Affiliation 1**.

“Department of Neurosurgery, Center for Neuroregeneration, Houston Methodist Research Institute, Houston, TX, United States” should be deleted, only “Russian Federation State Scientific Center—Institute of Biomedical Problems of the Russian Academy of Sciences, Moscow, Russia” should be kept.

The authors apologize for this error and state that this does not change the scientific conclusions of the article in any way. The original article has been updated.

